# Trends and projections of Hepatitis A incidence in eastern China from 2007 to 2021: an age-period-cohort analysis

**DOI:** 10.3389/fpubh.2024.1476748

**Published:** 2024-12-04

**Authors:** Hui Peng, Yin Wang, Songting Wei, Weili Kang, Xuefeng Zhang, Xiaoqing Cheng, Changjun Bao

**Affiliations:** ^1^GuSu Center for Disease Prevention and Control, Suzhou, China; ^2^Jiangsu Field Epidemiology Training Program, Jiangsu Provincial Centre for Disease Control and Prevention, Nanjing, China; ^3^Yangzhou Center for Disease Control and Prevention, Yangzhou, China; ^4^Heilongjiang Provincial Center for Disease Control and Prevention, Harbin, China; ^5^Jiangsu Provincial Centre for Disease Control and Prevention (Jiangsu Institution of Public Health), Nanjing, China

**Keywords:** Hepatitis A, age-period-cohort model, incidence trends, public health, Jiangsu Province, bayesian age-period-cohort model

## Abstract

**Objective:**

This study aimed to analyze the trends in Hepatitis A incidence associated with age, period, and birth cohorts from 2007 to 2021 in Jiangsu Province, China, and projects the future burden through 2031.

**Methods:**

Data on Hepatitis A cases in Jiangsu Province from 2007 to 2021 were obtained from the National Notifiable Disease Reporting System. Joinpoint regression analysis identified significant changes in incidence trends. The age-period-cohort model assessed the effects of age, period, and cohort on Hepatitis A incidence rates. Projections for 2022–2031 were generated using the Bayesian age-period-cohort model.

**Results:**

From 2007 to 2021, Hepatitis A incidence in Jiangsu Province significantly declined, with an average annual percent change (AAPC) of −10.77%. The decline was more pronounced in males (AAPC = −12.87%) compared to females (AAPC = −7.46%). The overall net drift was −10.61% (95% CI: −11.14% to −10.07%), the net drift for males was −12.77% (95% CI: −13.40% to −12.13%), which was higher than that for females at −7.27% (95% CI: −7.93% to −6.60%). The local drift indicates the incidence of hepatitis A decreased gradually, with the rate of decline slowing in the later period. Descriptive analysis revealed the highest incidence of Hepatitis A cases in the 40–59 age group, while age-period-cohort analysis indicated higher incidence rates in younger individuals. The cohort effect showed a continuous decline from the earliest cohort in 1923–1927 (Overall RR = 64.93, 95% CI: 42.55 to 99.07) to the most recent cohort in 1993–1997 (Overall RR = 0.008, 95% CI: 0.004 to 0.01). But Bayesian age-period-cohort model projections for 2022–2031 suggest that incidence rates will remain low, though they may slightly increase by 2031, with peak incidence shifting to the 60–64 age group.

**Conclusion:**

The incidence of Hepatitis A in Jiangsu Province has significantly decreased from 2007 to 2021, primarily due to public health measures and vaccination programs. Future efforts should focus on maintaining vaccination coverage and improving sanitation and hygiene practices to sustain these achievements.

## 1 Introduction

Hepatitis A, caused by the Hepatitis A virus (HAV), is an acute infectious disease that primarily affects the liver. It spreads mainly through the fecal-oral route and can also be transmitted via person-to-person contact, leading to large-scale outbreaks (1). Clinical symptoms include nausea, vomiting, fever, jaundice, and hepatosplenomegaly, with severe cases potentially resulting in liver failure. Although improved economic and sanitary conditions have led to an annual decrease in Hepatitis A incidence, WHO reports a 4% increase in global cases from 2010 to 2019, with frequent outbreaks in certain regions (2–4). Therefore, controlling Hepatitis A remains a global priority and a significant public health challenge for China.

The age-period-cohort (APC) model is a statistical tool used to analyze trends in disease incidence and mortality, as well as to project future changes in disease burden. It decomposes time trends into three effects: age, period, and cohort, providing more accurate estimates of long-term trends. The age effect reflects how different age stages influence disease risk; the period effect captures the impact of public health or social events in specific years; and the cohort effect indicates changes in disease rates due to varying environmental exposures among birth cohorts. APC analysis is widely used in cancer epidemiology (5, 6), chronic disease research (7), and infectious disease studies (8). Bayesian analysis offers a method for calculating the probability of hypotheses by combining prior information about unknown parameters with sample data using Bayes’ theorem, resulting in robust and reliable estimates. Consequently, the Bayesian age-period-cohort (BAPC) model is commonly used to project future age-specific disease incidence or mortality (9).

Previous studies have reported trends in the burden of Hepatitis A in China (10–12), but they have not detailed the specific contributions of age, period, and birth cohort to the overall trend, nor have they extensively explored future trends. This study aims to fill these gaps by analyzing the trend of Hepatitis A incidence in Jiangsu Province using Joinpoint regression and the APC model, and projecting future trends with the BAPC model, thereby providing new insights for Hepatitis A prevention and control.

## 2 Methods

### 2.1 Data source

Data on Hepatitis A cases in Jiangsu Province from 2007 to 2021 were obtained from the National Notifiable Disease Reporting System (NNDRS) of China. Reporting of Hepatitis A cases is mandatory and routine, theoretically allowing the NNDRS to capture all cases seeking medical consultation. Cases were filtered based on the date of onset and current address within Jiangsu Province, and duplicate cases with identical names, genders, and ages were removed to form the study database. Population data for Jiangsu Province from 2007 to 2021 were retrieved from the basic information system of NNDRS. Population projections for Jiangsu Province from 2022 to 2031 were generated based on the fixed ratio of Jiangsu’s population to that of China’s population.

### 2.2 Statistical analysis

Data were organized using excel 2019 and analyzed using Joinpoint regression software (V.5.0) and the APC model web analysis tool. BAPC model projections for Hepatitis A incidence from 2022 to 2031 were conducted using the BAPC package in R 4.3.3 software. Graphs were generated using GraphPad Prism 10.0 software. The significance level was set at a two-tailed *α* = 0.05, and all analyses compared differences between genders.

### 2.3 Ethical consideration

Ethical approval was not required for this study as it did not involve any identifiable personal information, such as names, identity information, addresses, or phone numbers.

### 2.4 Joinpoint regression

Joinpoint regression analysis was conducted using Joinpoint 5.0 software developed by the U.S. National Cancer Institute to determine long-term trends in Hepatitis A incidence, identify turning points, and calculate the Annual Percent Change (APC) and Average Annual Percent Change (AAPC). The case data were saved in .csv format and imported into the software. The Permutation Test was employed to determine statistically significant turning points. APC indicates changes over different time periods, whereas AAPC indicates changes over the entire study period. Positive APC and AAPC values indicate increasing trends, whereas negative values indicate decreasing trends. If there are no turning points, APC equals AAPC (13).

### 2.5 APC model

In this study, the APC model was employed to simultaneously explain the effects of age, period, and cohort on the incidence of Hepatitis A. The APC model is based on the Poisson distribution, with the general form of the equation as follows:

$$Y_{ijk}=\log\left(\lambda_{ijk}\right)=\mu +\alpha_{i}+\beta_{j}+\gamma_{k}+\varepsilon_{ijk}$$

where $Y_{ijk}$ is the outcome variable, $Y_{ijk}$ or $\log\left(\lambda_{ijk}\right)$ corresponds to the incidence of Hepatitis A in the $i^{th}$ age group, $j^{th}$ period, and $k^{th}$ cohort. *μ* is the intercept representing the overall average effect. The error $\varepsilon_{ijk}$ is assumed to be additive, independent, and normally distributed. *α*, *β*, and *γ* are the log-linear model coefficients corresponding to the age, period, and cohort effects, respectively. The usual constraint is that the sum of the parameters equals zero, expressed as $\sum \alpha_{i}=\sum \beta_{j}=\sum \gamma_{k}$ . The APC model requires equal time intervals for periods, ages, and cohorts; therefore, we selected individuals aged 20–84 grouped into 5-year intervals for analysis. This resulted in 13 age groups (20–24, 25–29, …, 80–84 years), 3 periods (2007–2011, 2012–2016, 2017–2021), and 15 cohorts (1923–1927, 1928–1932, …, 1993–1997). The tool developed by the National Cancer Institute was employed for parameter and effect estimation of the APC model^1^. The net drift, local drift, longitudinal age curve, and relative risk (RR) of period and cohort effects (using 2012–2016 as the reference period and 40–44 years as the reference age) were evaluated.

## 3 Result

### 3.1 Descriptive analysis and joinpoint regression analysis of Hepatitis A incidence in Jiangsu Province

**Figure 1:**
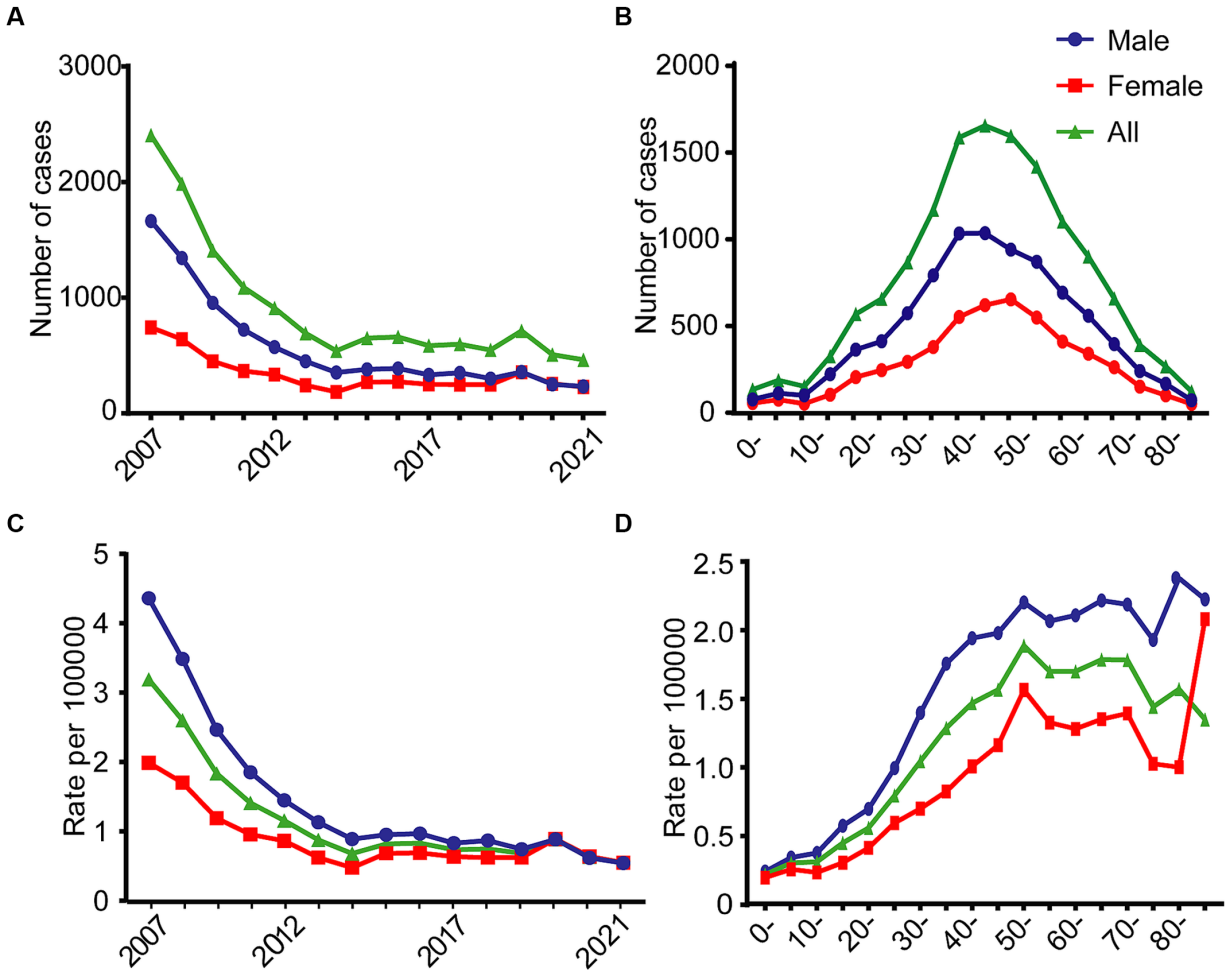
Changes in the number of cases and incidence rates of Hepatitis A in Jiangsu province, China, stratified by gender, from 2007 to 2021. **(A)** Annual number. **(B)** Age-specific number. **(C)** Annual incidence rate. **(D)** Age-specific incidence rate.

From 2007 to 2021, a total of 13,764 Hepatitis A cases were reported in Jiangsu Province, with a male-to-female ratio of 1.70:1. The average annual reported incidence rate was 1.16 per 100,000 population, with males exhibiting a higher incidence rate (1.45 per 100,000) compared to females (0.87 per 100,000). The overall incidence of Hepatitis A showed a declining trend during this period, with the gender disparity narrowing over time. Similar numbers of cases were reported for males and females from 2019 to 2021 (Figure 1A). The age distribution of cumulative cases from 2007 to 2021 showed a peak in the middle-aged groups, with the highest number of cases occurring in the 40–59 age range (Figure 1B). The trend in overall incidence rates paralleled the trend in the number of cases, with the total population incidence rate decreasing from 3.19 per 100,000 in 2007 to 0.55 per 100,000 in 2021. The gender ratio of incidence rates (male to female) decreased from 2.19:1 in 2007 to 0.99:1 in 2021 (Figure 1C). From 2007 to 2021, the incidence of Hepatitis A in Jiangsu Province gradually increased in all age groups from 0 to 54 years, then stabilized at those levels. The highest incidence rate was observed in the 50–54 age group (overall: 1.89 per 100,000; male: 2.20 per 100,000; female: 1.56 per 100,000) (Figure 1D).

**Figure 2:**
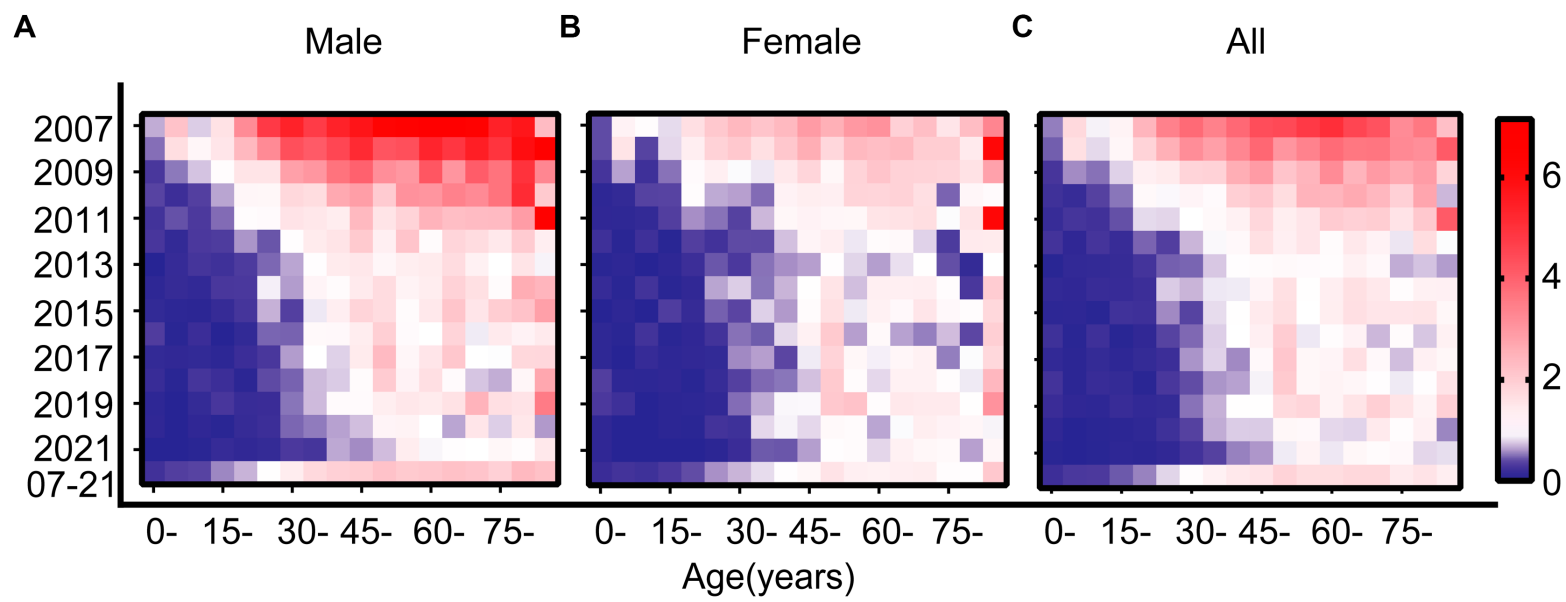
Lexis diagram of Hepatitis A incidence rates in Jiangsu province, China, from 2007 to 2021, stratified by year of diagnosis and age at diagnosis. **(A)** Males. **(B)** Females. **(C)** Overall. In each diagram, blue represents lower incidence rates, and red represents higher incidence rates. The scale is consistent across all panels. For each panel: rows indicate the incidence rates across specific age groups within a given calendar year; columns indicate the incidence rates within specific age groups across different calendar years.

A detailed comparison of the incidence rates across different age groups for both genders from 2007 to 2021 is shown in the Lexis diagram (Figure 2). Each row shows the incidence rates for different age groups over the years for both males and females. The diagram indicated a general decline in incidence rates across most age groups. Each column shows the incidence rates for different years over the age groups. When comparing across different age groups along each column of the Lexis diagram, it was evident that younger age groups have lower incidence rates of Hepatitis A, and the peak age of onset in females shifts earlier than in males.

**Figure 3:**
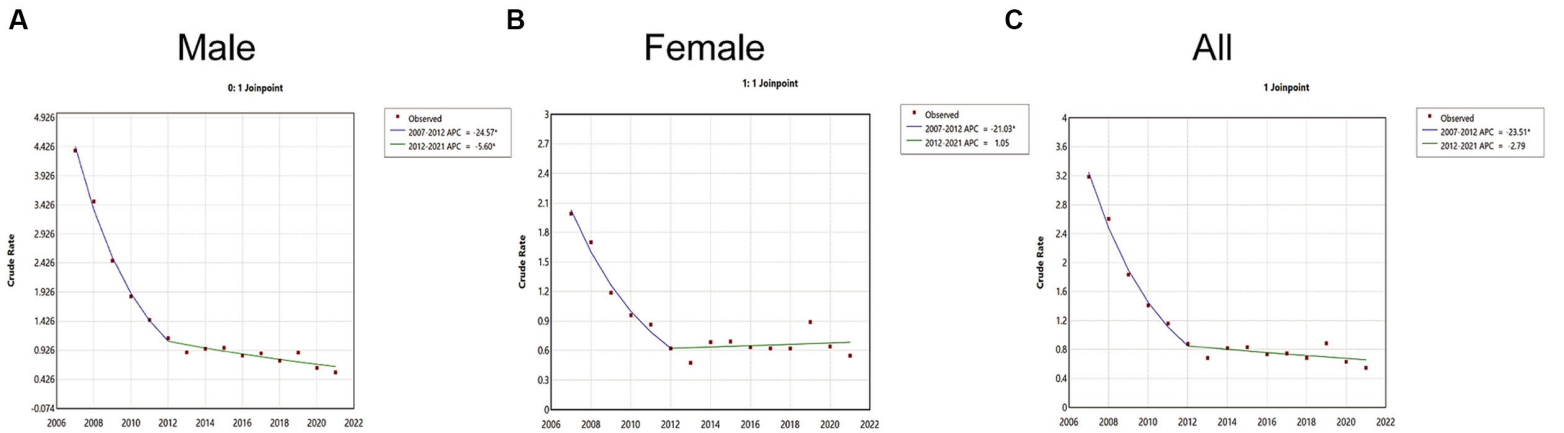
Joinpoint regression model of Hepatitis A incidence rates in Jiangsu Province, China, from 2007 to 2021. **(A)** Males. **(B)** Females. **(C)** Overall.

The age-specific incidence rates for both males and females, as well as the overall population, exhibited a consistent downward trend, each with a single joinpoint (Joinpoint = 1). The overall incidence rate of Hepatitis A from 2007 to 2021 showed a significant decline (AAPC = −10.77, 95% CI: −12.27 to −9.55, *p* < 0.05). During the period from 2007 to 2012, there was a rapid decline (APC = −23.51, *p* < 0.05), followed by a stable period from 2012 to 2021 (APC = −2.79, *p* > 0.05) (Figure 3C). The decline in Hepatitis A incidence rates was most pronounced among males (AAPC = −12.87, 95% CI: −14.31 to −11.81, *p* < 0.05). For males, there was a rapid decline from 2007 to 2012 (APC = −24.57, *p* < 0.05) and a slower decline from 2012 to 2021 (APC = −5.60, *p* < 0.05) (Figure 3A). For females, the decline in Hepatitis A incidence rates was smaller (AAPC = −7.46, 95% CI: −9.18 to −5.92, *p* < 0.05). The incidence rate for females showed a rapid decline from 2007 to 2012 (APC = −21.03, *p* < 0.05) and then stabilized from 2012 to 2021 (APC = −1.05, *p* > 0.05) (Figure 3B).

### 3.2 APC model analysis of Hepatitis A incidence rates

**Figure 4:**
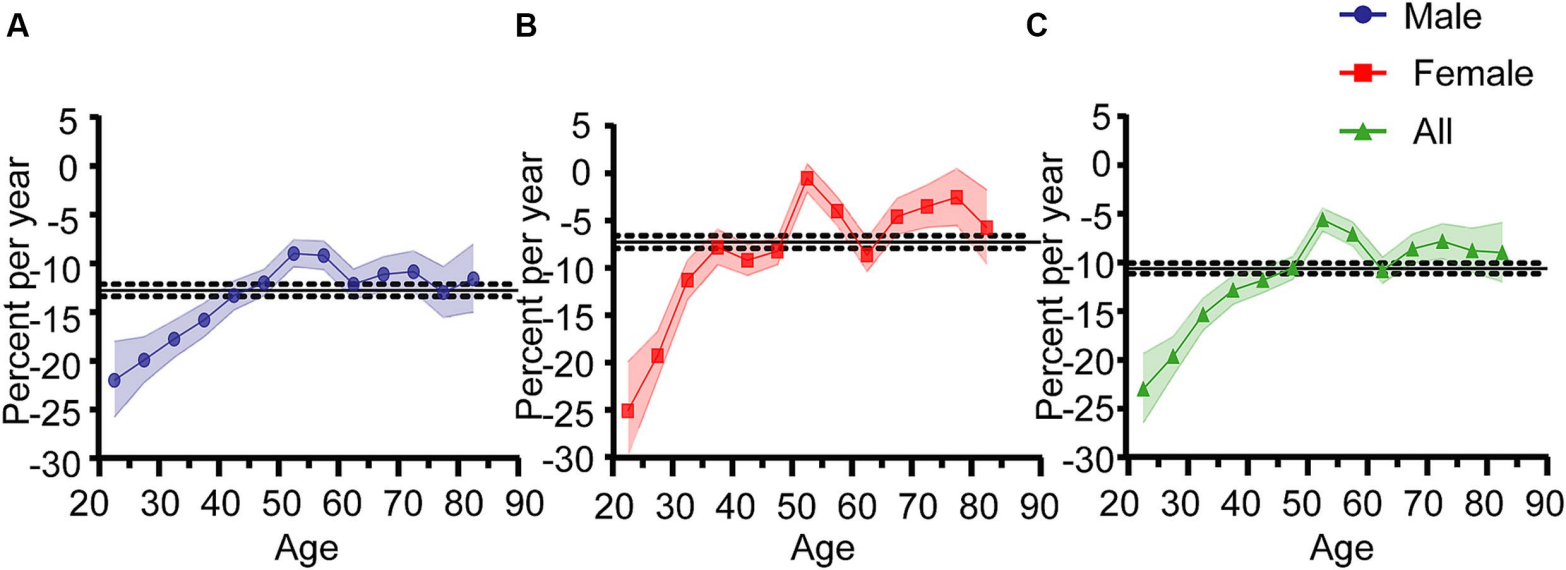
Net drift and local drift of Hepatitis A incidence rates in Jiangsu Province, China, from 2007 to 2021. **(A)** Males. **(B)** Females. **(C)** Overall. The horizontal black solid line represents the net drift value, and the black dashed lines correspond to the 95% confidence intervals (CI). The colored solid lines represent the local drift values, with the colored bands showing the 95% CI.

**Figure 5:**
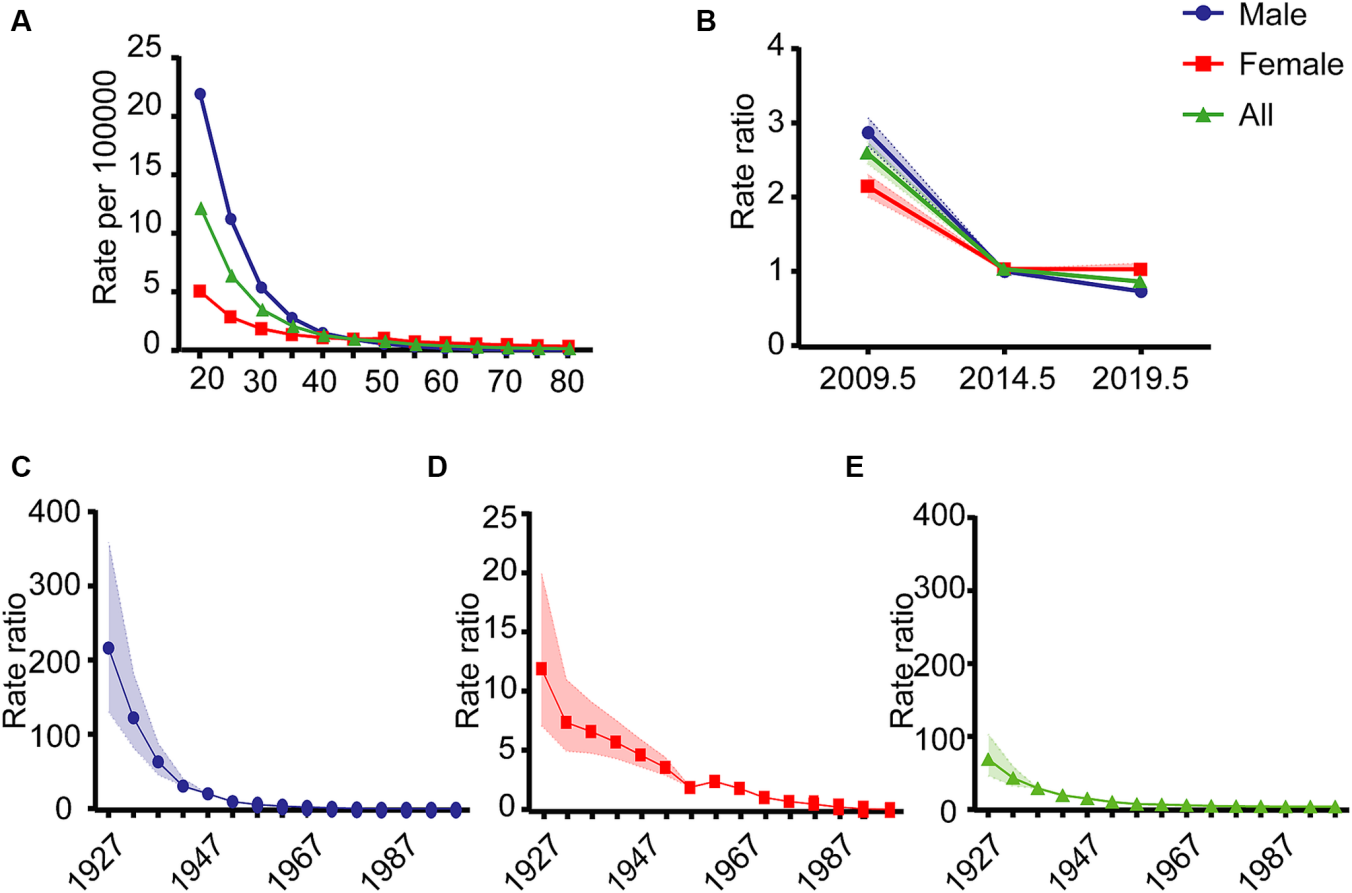
Longitudinal age curves, period effects, and cohort effects of Hepatitis A in Jiangsu Province, China, from 2007 to 2021. **(A)** Longitudinal age curves. **(B)** Period effects. **(C)** Cohort effect for males. **(D)** Cohort effect for females. **(E)** Overall cohort effect.

The estimated effects of age, period, and cohort on Hepatitis A incidence rates are illustrated in Figure 4. The net drift represents the annual percentage change in expected age-adjusted rates over time, indicating the overall trend for specific age groups. Local drift refers to the annual percentage change in specific age groups during a particular period. From 2007 to 2021, the overall net drift for Hepatitis A incidence rates was −10.61% (95% CI: −11.14% to −10.07%). The net drift for males was −12.77% (95% CI: −13.40% to −12.13%), which was higher than that for females at −7.27% (95% CI: −7.93% to −6.60%). Overall local drift values were generally less than zero, local drift values for different age groups ranged from −22.98% (95% CI: −26.45% to −19.34%) to −5.61% (95% CI: −6.76% to −4.43%). For females, local drift values ranged from −25.12% (95% CI: −29.98% to −19.93%) to −0.51% (95% CI: −1.99 to 0.98%), indicating greater variability compared to males, whose local drift values ranged from −21.99% (95% CI: −25.79% to −18.00%) to −8.97% (95% CI: −10.33% to −7.60%). The absolute values of local drift for both genders and overall exhibited a monotonic decrease from age 20 to 45. For ages 45 to 84, an M-shaped local drift curve was observed, with absolute local drift values being less than the net drift. The lowest absolute local drift value was observed in the 50–54 age group. The longitudinal age curve, which adjusts for period effects, represents age-related disease trends within specific cohorts. This curve also shows that after adjusting for period and cohort effects, Hepatitis A incidence rates decrease in a “rapid-slow” pattern with increasing age (Figure 5A).

The period effect represents the relative risk of disease incidence in each period compared to a reference period, after adjusting for age and cohort nonlinear effects. Similarly, the cohort effect represents the relative risk of disease incidence for each birth cohort compared to a reference cohort, after adjusting for age and period nonlinear effects. These effects help infer trends in disease incidence related to specific periods and cohorts. The period effect showed a “rapid decline-slow decline” trend for the overall population and males, while for females, it showed a “rapid decline-stable” trend (Figure 5B). The relative risk (RR) of incidence rates was highest in the period 2007–2011 when compared to the reference period. The decline in the RR for males was generally faster than for females (Overall RR = 2.57, 95% CI: 2.43 to 2.71; Male RR = 2.87, 95% CI: 2.68 to 3.07; Female RR = 2.12, 95% CI: 1.97 to 2.27).

The pattern of cohort effects was similar to that of period effects. The cohort effect showed a continuous decline from the earliest cohort in 1923–1927 (Overall RR = 64.93, 95% CI: 42.55 to 99.07) to the most recent cohort 1993–1997 (Overall RR = 0.008, 95% CI: 0.004 to 0.01). The decline in the cohort effect was not uniform, with incidence rates declining faster for males than for females. As birth years increased, the incidence rates for males and females tended to converge (Figures 5C–E). Additionally, the Wald χ^2^ test indicated that the net drift, local drift, period, and cohort effects were all statistically significant (*p* < 0.05) (Table 1).

**Table 1:** The Wald’s Chi-square tests for the estimated parameters of the age-period-cohort model.

Gender	Null hypothesis	*χ^2^*	*df.*	*P*-value
Overall	Net drift = 0	1346.37	1	<0.001
	All age deviations = 0	49.98	11	<0.001
	All period *RR* = 1	180.52	1	<0.001
	All cohort *RR* = 1	227.69	13	<0.001
	All local drifts = net drift	1850.02	2	<0.001
Male	Net drift = 0	1352.16	1	<0.001
	All age deviations = 0	21.67	11	0.027
	All period *RR* = 1	119.56	1	<0.001
	All cohort *RR* = 1	125.14	13	<0.001
	All local drifts = net drift	1850.86	2	<0.001
Female	Net drift = 0	422.18	1	<0.001
	All age deviations = 0	75.59	11	<0.001
	All period *RR* = 1	116.57	1	<0.001
	All cohort *RR* = 1	237.93	13	<0.001
	All local drifts = net drift	614.57	2	<0.001

### 3.3 Bayesian age-period-cohort projection of Hepatitis A incidence rates

**Figure 6:**
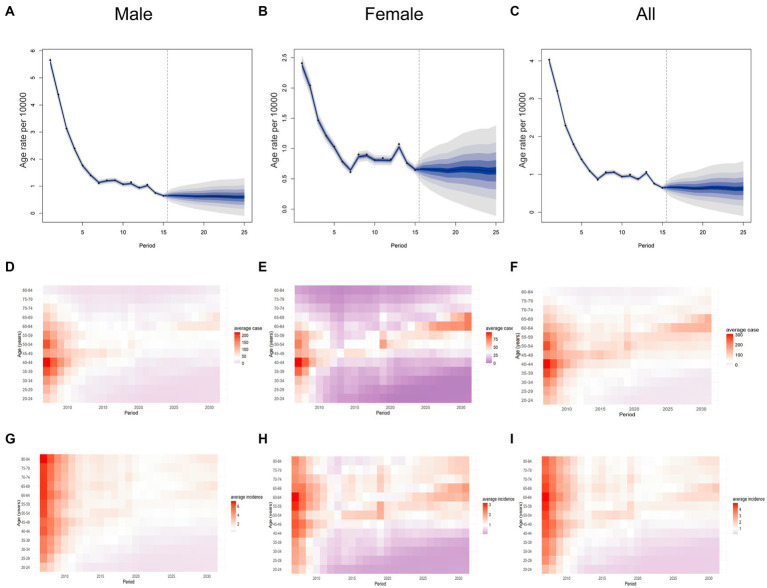
Projected Number of Hepatitis A Cases and Incidence Rates in Jiangsu Province, China, by Age group for 2022–2031. **(A)** incidence rate for males. **(B)** incidence rate for females. **(C)** overall incidence rate. **(D)** Annual number of cases for males. **(E)** Annual number of cases for females. **(F)** Annual overall number of cases. **(G)** Annual incidence rate for males. **(H)** Annual incidence rate for females. **(I)** Annual overall incidence rate.

The BAPC model was employed to forecast the number of Hepatitis A cases and incidence rates in Jiangsu Province, China, from 2022 to 2031. By 2031, it was projected that there will be 688 cases of Hepatitis A, representing 0.29 times the number of cases in 2007 and 1.44 times the number in 2021. These projections demonstrate heterogeneity across different age groups. For the overall population and both genders, the number of Hepatitis A cases was anticipated to continue declining in age groups under 60. However, for females aged 60 and above, the number of cases was expected to increase. Among males, the number of cases was anticipated rising in the 60–69 age group while remaining stable at low levels for those aged 70 and above (the instrument can be found in Supplementary Tables S1–S3). The overall incidence of Hepatitis A was expected to remain relatively low from 2022 to 2031 (Figure 6). By 2031, the incidence rate in Jiangsu Province is projected to be 0.94 per 100,000 population, representing 0.42 times the rate in 2007 and 1.46 times the rate in 2021. For males, the projected incidence rate was 0.93 per 100,000, representing 0.17 times the rate in 2007 and 1.40 times the rate in 2021. For females, the incidence rate was projected to be 0.95 per 100,000, representing 0.42 times the rate in 2007 and 1.52 times the rate in 2021. The age group with the highest projected incidence in 2022–2031 was the 60–64 age group, with a rate of 1.49 per 100,000, within this group, the incidence rate for females is projected to be 1.17 times higher than that for males.

## 4 Discussion

Our study employed Joinpoint regression analysis to evaluate trends in Hepatitis A incidence from 2007 to 2021. Subsequently, we applied the APC model to describe and analyze the impacts of age, period, and cohort on Hepatitis A incidence in the 20–84 age group in Jiangsu, China. Furthermore, we utilized the integrated nested Laplace approximation technique to estimate the BAPC model, projecting Hepatitis A incidence rates from 2022 to 2031. Our findings revealed that from 2007 to 2021, the net drift value was negative, indicating a gradual decrease in Hepatitis A incidence in Jiangsu, although the rate of decline slowed over time. Both local drift and longitudinal age curves demonstrated a rapid-to-slow decline in incidence rates, indicating that younger individuals are a high-risk group for prioritized interventions. The APC analysis indicated that the period effect decreased from 2007 to 2021, suggesting that local Hepatitis A vaccination strategies and other public health measures have been effective over the past decades. Additionally, the declining cohort effect implied a reduction in epidemiological risk exposure to Hepatitis A. The BAPC model projected that the incidence of Hepatitis A will remain low by 2031 but will be slightly higher than in 2021, with the peak age of incidence projected to shift to 60–64 years.

Descriptive analysis indicated that the average incidence rate of Hepatitis A in Jiangsu Province remained at a low endemic level (incidence rate < 4/100,000) from 2007 to 2021, showing a yearly decline. This trend is consistent with those observed in other provinces in China, such as Beijing (11) and Zhejiang (12). From 2005 to 2014, the reported incidence rate of hepatitis E in Jiangsu Province exhibited a relatively stable trend, while the proportion of reported viral hepatitis cases increased annually (14). Although HAV and HEV are transmitted through the gastrointestinal tract, the specific transmission route of HAV is clearer than that of HEV, HAV is limited to humans and is primarily transmitted via the fecal-oral route, whereas HEV can also be transmitted zoonotically; and the HEV vaccine was officially launched in 2012, but it has not been widely used. This discrepancy may explain the differing long-term trends of HAV and HEV (15, 16). The higher incidence among males may be attributed to greater exposure opportunities due to social activities and poorer hygiene habits. The high incidence age group was 40–59 years, possibly because symptoms in children are often less noticeable. As individuals age, the symptoms become more apparent and are more likely to be diagnosed. Additionally, adults have a wider social range, thereby increasing their chances of contracting Hepatitis A (11, 17). Furthermore, older adults exhibited a higher incidence rate, primarily due to a smaller population base, making them an important group to consider for interventions. The gender differences in the curve may result from different lifestyles, with males having broader social networks and higher exposure opportunities, including behaviors such as men who have sex with men (MSM) (18–20). The male-to-female ratio of Hepatitis A incidence decreased annually, gradually leveling out, likely due to increased awareness and education on disease prevention, improved urban hygiene, and better public health practices. However, the Lexis diagram indicated higher incidence rates in middle-aged and older adults compared to younger individuals. In contrast, the APC model revealed the true age pattern of Hepatitis A incidence, differing from that shown in the Lexis diagram.

The age effect represents the differences in incidence rates among various age groups, potentially influenced by complex factors such as personal health status and lifestyle behaviors (21). The longitudinal age curve illustrates age-specific temporal trends, and its pattern was consistent with studies from Serbia and South Korea (22, 23), indicating that young people are a high-risk group for Hepatitis A incidence. The curve shows the highest incidence rate among the 20–34 age group, likely due to lower vaccination coverage for Hepatitis A and a high level of social activity, both of which increase the risk of infection. Another possible reason is the continued decline in Hepatitis A incidence in recent years, which reduces natural infection opportunities and potentially leads to lower herd immunity levels against HAV among young and middle-aged people. Additionally, studies show that long-term immunity can develop after Hepatitis A virus infection, reducing the incidence rate risk in older age groups (24). The local drift distribution shows an M-shaped pattern, initially rising and then fluctuating. Throughout the entire period, the incidence rate of Hepatitis A has declined, with a faster decline observed in younger age groups. Therefore, this finding suggests prioritizing public health interventions for the 20–30 age group to achieve better control outcomes.

The period effect is influenced by external macro factors such as social, economic, medical, and policy changes, which affect the health status of the entire society or population. This study shows a continuous decline in the period effect over the past 15 years. The incidence of Hepatitis A is closely linked to socioeconomic indicators and access to safe drinking water. In China, with increased income and improved access to clean water, along with the promotion of various health policies such as the Patriotic Health Campaign and the improvement of water and sanitation facilities, public health knowledge has gradually improved, thereby reducing the incidence of Hepatitis A (25, 26). Since 2005, the NNDRS system has utilized a modern real-time network information system to report and register cases, enabling timely tracking and management, thereby effectively reducing the spread of cases. Additionally, since 2007, China has included the Hepatitis A vaccine in the national immunization program, administering a single dose of live attenuated Hepatitis A vaccine to children at 18 months of age. After the introduction of Hepatitis A vaccine in Shaanxi, Henan and Beijing of China, the incidence of reported cases of Hepatitis A has decreased significantly (11, 27, 28), other countries, such as Israel, Bahrain and other regions (Australia, Belarus, Italy, Spain, United States), saw a sharp decline in disease incidence not only in vaccinated cohorts but also in the entire population within the years after the start of mass vaccination (29). Follow-up data show that 68% of subjects maintain immunity for 20 years (30). There is also evidence that the immune memory induced by the Hepatitis A vaccine persists even after detectable antibodies are lost (31), indicating that the vaccine effectively reduces the incidence of Hepatitis A. However, some studies have raised concerns that vaccine-induced immunity differs from immunity acquired through natural infection, and we still do not know what will happen 20 or even 50 years after vaccination. A 2014 seroepidemiological study found a declining trend in antibodies among vaccinated children, with an anti-HAV positive rate of only 61.97% in the 10–14 age group, indicating that the durability of the vaccine may not be sufficient. If vaccine-induced immunity wanes as vaccinated children reach adulthood, outbreaks may occur, and the symptoms in adults can be more severe than in children, thereby increasing the disease burden.

The cohort effect refers to the different disease incidence rates among various generations and birth cohorts associated with specific epidemiological exposures, such as wars, famines, baby booms, and pandemics. This intergenerational effect can significantly impact the course of life. Our study revealed higher RR for earlier cohorts. The observed differences and reductions in the cohort effect may be attributed to China’s gradually stabilizing socioeconomic status, rapid development, continuous economic improvement, and enhanced quality of life. These factors have led to reduced epidemiological risk exposure to Hepatitis A in later birth cohorts. Early infection with Hepatitis A can generate long-lasting protective antibodies, potentially forming a herd immunity barrier and thereby reducing the exposure risk for later birth cohorts. A herd immunity barrier can be established when the population antibody positivity rate exceeds 80%. A 1994 study in China found that the population HAV IgG antibody positivity rate was 85.67% (32).

The BAPC model projections indicate that the incidence rate of Hepatitis A will remain low from 2022 to 2031. However, there will be a slight increase compared to 2021, with the peak age of incidence gradually shifting to older age groups. Historically, the high-risk group for Hepatitis A incidence has been young children. Since China included Hepatitis A vaccination in the national immunization program in 2007, it has significantly protected individuals under 20 years old (11). In middle-income countries with rising incomes, rapid urbanization, and nearly universal access to clean water, particularly in urban areas, the incidence of Hepatitis A is declining, and the median age of infection is increasing. The peak age of incidence in China is gradually shifting to older age groups, similar to the epidemiological patterns observed in many countries. From 1990 to 2005, the age at the midpoint of population immunity (AMPI) significantly increased in East Asia, Southeast Asia, Eastern and Central Europe, Latin America, and parts of the Middle East and North Africa (25, 33). And from 2022 to 2031, projection of the incidence rate among the 20–39 age group is expected to remain low, suggesting that the vaccine provides substantial protection. This aligns with Chinese studies indicating that the vaccine offers protection for at least 20 years (34). Widespread childhood vaccination against Hepatitis A is a cost-effective public health intervention. However, initially lower incidence rates of Hepatitis A can lead to increased hospitalizations and deaths. The generation of susceptible individuals with long-term low exposure to the virus face a higher risk of symptomatic Hepatitis A in old age. Older patients with Hepatitis A often experience much more severe symptoms than infected children. Lower incidence rates can delay the typical age of infection, increasing symptomatic cases in the population (35). Additionally, the incidence rate among women is projected to gradually increase from 2022 to 2031, likely due to women’s relatively longer lifespan and the larger number of older women compared to men. These projections indicate that while the overall incidence of Hepatitis A in Jiangsu Province is anticipated to remain low, targeted public health interventions may be needed to address the increasing incidence rates in older age groups, particularly among females.

Increasing economic interdependence, social integration, and other aspects of globalization are contributing to significant changes in the epidemiology of Hepatitis A. Our study employs the APC model to quantify the age, period, and cohort effects, and to analyze their potential underlying causes. To summarize, the primary measures implemented to reduce the incidence of hepatitis A over the past 15 years include: first, enhancing the safety of drinking water and food to eradicate transmission at its source; second, improving the capacity of medical personnel to accurately identify and differentiate infected individuals for early detection, diagnosis, and reporting; third, increasing vaccination coverage among children and other vulnerable populations; and fourth, enhancing public awareness and educational initiatives to promote disease prevention and encourage informed vaccination. Although the incidence rate of HAV infection in China is gradually decreasing, the proportion of symptomatic cases is increasing with the average age of infection. Conducting population immunity antibody testing can help identify high-risk groups for targeted vaccination.

## 5 Conclusion

The significant decline in Hepatitis A incidence in Jiangsu Province from 2007 to 2021 can be primarily attributed to public health measures and vaccination programs. The APC model highlighted the importance of age, period, and cohort factors in understanding the epidemiology of Hepatitis A. To sustain these gains, future efforts should focus on maintaining vaccination coverage and improving sanitation and hygiene practices. Additionally, targeted public health interventions are necessary to address the increasing incidence rates in older age groups, particularly among females. Continuous monitoring and timely interventions are essential to maintaining control over Hepatitis A in Jiangsu Province.

## Data Availability

The original contributions presented in the study are included in the article/Supplementary material, further inquiries can be directed to the corresponding authors.

## Footnotes

^1^https://analysistools.nci.nih.gov/apc/
