# Supplementary material for: Trends and projections of Hepatitis A incidence in eastern China from 2007 to 2021: an age-period-cohort analysis
Source: Front Public Health. 2024 Dec 4;12:1476748. doi: 10.3389/fpubh.2024.1476748 (PMC11652658; doi:10.3389/fpubh.2024.1476748)
Supplement: Supplementary file 1 [file Table_1.docx]

Supplementary Material

# Supplementary Tables

**1.1 Supplementary Table 1.** The projected age-specific number of Hepatitis A by the Bayesian age-period-cohort model from 2022 to 2031 in Jiangsu, China (overall).

| Age group | Calendar year | | | | | | | | | | |
| --- | --- | --- | --- | --- | --- | --- | --- | --- | --- | --- | --- |
|  | 2022 | 2023 | 2024 | 2025 | 2026 | 2027 | 2028 | 2029 | 2030 | 2031 | Row total |
| 20-24 | 4 | 4 | 5 | 5 | 5 | 5 | 6 | 6 | 6 | 7 | 54 |
| 25-29 | 6 | 6 | 5 | 5 | 5 | 5 | 5 | 6 | 6 | 6 | 56 |
| 30-34 | 17 | 13 | 10 | 9 | 7 | 7 | 6 | 6 | 6 | 5 | 85 |
| 35-39 | 23 | 25 | 27 | 27 | 23 | 19 | 15 | 12 | 10 | 8 | 188 |
| 40-44 | 33 | 32 | 30 | 28 | 29 | 30 | 33 | 36 | 36 | 31 | 318 |
| 45-49 | 54 | 55 | 53 | 50 | 51 | 55 | 53 | 50 | 47 | 49 | 516 |
| 50-54 | 77 | 75 | 69 | 65 | 67 | 68 | 69 | 67 | 64 | 65 | 686 |
| 55-59 | 84 | 85 | 86 | 88 | 87 | 88 | 87 | 80 | 75 | 78 | 837 |
| 60-64 | 51 | 67 | 87 | 100 | 127 | 134 | 135 | 136 | 140 | 138 | 1114 |
| 65-69 | 54 | 52 | 46 | 44 | 46 | 59 | 78 | 101 | 117 | 148 | 746 |
| 70-74 | 40 | 42 | 47 | 51 | 55 | 60 | 58 | 52 | 49 | 51 | 505 |
| 75-79 | 23 | 27 | 33 | 36 | 39 | 41 | 43 | 49 | 54 | 58 | 403 |
| 80-84 | 15 | 16 | 18 | 21 | 22 | 26 | 29 | 36 | 40 | 43 | 266 |
| Column total | 481 | 498 | 515 | 529 | 563 | 597 | 617 | 635 | 649 | 688 | 5773 |

**1.2 Supplementary Table 2.** The projected age-specific number of Hepatitis A by the Bayesian age-period-cohort model from 2022 to 2031 in Jiangsu, China (male).

| Age group | Calendar year | | | | | | | | | | |
| --- | --- | --- | --- | --- | --- | --- | --- | --- | --- | --- | --- |
|  | 2022 | 2023 | 2024 | 2025 | 2026 | 2027 | 2028 | 2029 | 2030 | 2031 | Row total |
| 20-24 | 3 | 3 | 3 | 4 | 4 | 4 | 4 | 5 | 5 | 5 | 41 |
| 25-29 | 5 | 4 | 4 | 4 | 4 | 4 | 4 | 5 | 5 | 5 | 45 |
| 30-34 | 10 | 9 | 7 | 6 | 5 | 5 | 5 | 5 | 5 | 5 | 62 |
| 35-39 | 13 | 14 | 15 | 16 | 14 | 12 | 10 | 8 | 7 | 6 | 117 |
| 40-44 | 18 | 17 | 15 | 15 | 16 | 17 | 19 | 20 | 21 | 19 | 177 |
| 45-49 | 28 | 27 | 27 | 25 | 27 | 28 | 26 | 23 | 22 | 24 | 257 |
| 50-54 | 34 | 35 | 33 | 31 | 31 | 32 | 32 | 31 | 29 | 31 | 320 |
| 55-59 | 37 | 38 | 38 | 40 | 37 | 39 | 39 | 37 | 35 | 35 | 375 |
| 60-64 | 23 | 31 | 39 | 47 | 58 | 58 | 59 | 60 | 62 | 58 | 495 |
| 65-69 | 27 | 27 | 25 | 22 | 22 | 26 | 34 | 44 | 52 | 65 | 344 |
| 70-74 | 20 | 21 | 22 | 25 | 26 | 30 | 30 | 27 | 25 | 25 | 251 |
| 75-79 | 12 | 14 | 17 | 18 | 20 | 20 | 21 | 23 | 25 | 27 | 196 |
| 80-84 | 7 | 7 | 8 | 9 | 10 | 11 | 13 | 16 | 18 | 19 | 120 |
| Column total | 239 | 246 | 254 | 262 | 274 | 287 | 296 | 304 | 312 | 325 | 2798 |

**1.3 Supplementary Table 3.** The projected age-specific number of Hepatitis A by the Bayesian age-period-cohort model from 2022 to 2031 in Jiangsu, China (female).

| Age group | Calendar year | | | | | | | | | | |  |
| --- | --- | --- | --- | --- | --- | --- | --- | --- | --- | --- | --- | --- |
|  | 2022 | 2023 | 2024 | 2025 | 2026 | 2027 | 2028 | 2029 | 2030 | 2031 | Row total | |
| 20-24 | 1 | 1 | 2 | 1 | 1 | 1 | 2 | 1 | 1 | 2 | 13 |  |
| 25-29 | 1 | 2 | 1 | 1 | 1 | 1 | 1 | 1 | 1 | 1 | 11 |  |
| 30-34 | 7 | 4 | 3 | 3 | 2 | 2 | 1 | 1 | 1 | 0 | 23 |  |
| 35-39 | 10 | 11 | 12 | 11 | 9 | 7 | 5 | 4 | 3 | 2 | 71 |  |
| 40-44 | 15 | 15 | 15 | 13 | 13 | 13 | 14 | 16 | 15 | 12 | 141 |  |
| 45-49 | 26 | 28 | 26 | 25 | 24 | 27 | 27 | 27 | 25 | 25 | 259 |  |
| 50-54 | 43 | 40 | 36 | 34 | 36 | 36 | 37 | 36 | 35 | 34 | 366 |  |
| 55-59 | 47 | 47 | 48 | 48 | 50 | 49 | 48 | 43 | 40 | 43 | 462 |  |
| 60-64 | 28 | 36 | 48 | 53 | 69 | 76 | 76 | 76 | 78 | 80 | 619 |  |
| 65-69 | 27 | 25 | 21 | 22 | 24 | 33 | 44 | 57 | 65 | 83 | 402 |  |
| 70-74 | 20 | 21 | 25 | 26 | 29 | 30 | 28 | 25 | 24 | 26 | 254 |  |
| 75-79 | 11 | 13 | 16 | 18 | 19 | 21 | 22 | 26 | 29 | 31 | 207 |  |
| 80-84 | 8 | 9 | 10 | 12 | 12 | 15 | 16 | 20 | 22 | 24 | 146 |  |
| Column total | 242 | 252 | 261 | 267 | 289 | 310 | 321 | 331 | 337 | 363 | 2975 |  |
